# Access to City Center: Automobile vs. Public Transit

**DOI:** 10.3390/ijerph19095622

**Published:** 2022-05-05

**Authors:** Linlin Liu, Bohong Zheng, Chen Luo, Komi Bernard Bedra, Francis Masrabaye

**Affiliations:** School of Architecture and Art, Central South University, Changsha 410018, China; divine@csu.edu.cn (L.L.); luochenlc@csu.edu.cn (C.L.); komibedra@csu.edu.cn (K.B.B.); mfrancis@csu.edu.cn (F.M.)

**Keywords:** city center accessibility, online map, isochrone maps, cumulative opportunities, GWR

## Abstract

For current territory development planning in China, city center accessibility (CCA) has gained increasing attention for evaluating the expansion of urban areas. How should CCA and its differences between the automobile and public transit (PT) modes be measured? We analyzed CCA from travel time and travel cost perspectives using the travel data obtained from the Baidu Map at a 100 m × 100 m resolution. The GWR was then examined to explore the correlation between the explanatory variables and the CCA differences. Automobile-based CCA shows a concentric structure and varies with time, while PT-based CCA has an apparent linear expansion along the metro lines and fluctuates less. When measuring by travel cost instead of travel time, CCA gaps between the two modes are lessened, and the automobile’s advantage is no longer evident. The distance from the metro stations has a significant positive effect on CCA differences, and the positive effect concentrates in the 3.6 km range (measured by travel time) and 2.8 km range (measured by travel cost) around the metro stations. Our study highlights the importance of multiple perspectives when comparing the accessibility of different transport modes, and the results also provide implications for policy-makers.

## 1. Introduction

China’s State Council released the “National Main-function Area Plan” in 2010, which is the first territory development plan in China. In shifting from urban and rural planning to territory development planning, the government has issued various guidelines to guide the construction and control of land use. Within these guidelines, city center accessibility (CCA) gains more and more attention as an important indicator for evaluating the development of urban space and land use. Since Hansen [1] proposed the concept of accessibility, it has been defined in different ways, as accessibility is studied in various fields, such as socioeconomics, transportation, and urban planning [2]. Some researchers defined accessibility as “the ease and the ability to reach opportunities, activities, and services using a transport mode or a combination of modes” [3,4,5] to explain the interaction between land use and transport systems. CCA can be delineated as the ease of reaching the city center and measured by the time or the cost incurred in travel from the origin to the city center.

The ease of reaching city centers has a significant impact on urban residents’ lives in China because they are important commercial hubs, major destinations for business, consumption, and entertainment. With the acceleration of urbanization and the expansion of urban areas, the residential land spreads outwards, and the disparities of CCA among residents at different locations also increase. Public transport (PT) is considered to play a critical role in reducing private car usage, alleviating traffic pressure, ensuring social equity and sustainable development because of its higher energy efficiency and its lower cost for users [6,7]. Nowadays, more and more people advocate green travel, and urban public transport infrastructure is becoming perfected, further changing CCA’s distribution disparities. Equitable PT distribution can reduce the accessibility gap between different groups [8], because disadvantaged social groups have limited travel options and tend to rely more on PT to access opportunities [9]. According to Changsha’s 2018 resident travel survey report, the PT mode share is 54%, and this proportion is still increasing as more metro lines are being opened. One way to evaluate PT service equity is to understand the accessibility difference between PT and automobile. The comparison between the two modes provides information about the efficiency of PT, as better PT accessibility can affect PT travel choice, then generate higher PT mode share [10]. Increasing accessibility to urban services is crucial for transport policy and urban planning [11].

Accessibility difference between transport modes is not a new concept. In early studies comparing the accessibility of PT and automobile, PT was mainly focused on the bus mode. These studies point out that automobiles have better accessibility than PT [12,13,14]. Nowadays, the improvement of urban metro systems has brought new changes to the accessibility difference between PT and automobile. Related studies should include bus, metro, and a combination of both. However, when referring to accessibility compared between different transport modes, most accessibility measures only use time (or distance) and neglect the travel cost [15]. Such an analysis may be disadvantageous for PT and tends to underestimate its accessibility, as it has a more significant advantage in travel cost instead of travel time than automobile. In this paper, we convert travel time to its monetary value and add the actual travel fare to get the total travel cost, which reflects the monetary and non-monetary costs, to explore the following questions from travel time and travel cost perspectives.

- What are the differences between PT-based CCA and automobile-based CCA?
- How should these differences be measured?
- What are the variables that may influence these differences?

To answer the first two questions, by using the real-time travel data obtained from the online map, we calculate and visualize PT-based and automobile-based CCA separately, then the PT-based and automobile-based CCA ratio for each unit to describe the difference between the two modes. As for the third question, we examine the ordinary least squares (OLS) regression and the geographically weighted regression (GWR). This study aims to measure automobile-based and PT-based CCA under real transport networks without the tedious building process of road networks, trying to get more accurate and reasonable results. Because of the real-time characteristics of the acquired travel data, we can analyze CCA not only in spatial dimensions but also capture variations along temporal dimensions. Our research findings could aid in making policies that reduce socio-spatial inequalities.

The remainder of this article starts with a review of related research in Section 2. Section 3 describes the study area, data assembled and methods adopted. Then we present the results and analyses in Section 4, and conclusions are given in Section 5.

## 2. Literature Review

Since accessibility was proposed, various measures have been developed, such as gravity-based measures [1], the cumulative opportunities method [16,17], and the two-step floating catchment area method [18]. Accessibility is often measured by counting the number of opportunities reached within a given constraint, such as time or distance, usually combined with isochrone maps. Time measures can be more sensitive than distance measures when mirroring demographic, social, economic, and cultural constraints [19]. Therefore, accurate travel time estimation is crucial for measuring spatial accessibility [20]. In many studies, the travel time is often achieved with the help of network analysis tools in ArcGIS. Often, researchers first build a road network and set appropriate speeds for different levels of roads, then get the travel time of each road by dividing the road length by the speed [21,22]. Travel time calculated by this method is too ideal, and the considerable spatial variations are concealed. It is also challenging to embody the dynamic characteristics of accessibility for seldom considering the actual traffic status. Accessibility is not only a spatial concept but also contains temporal characteristics [23]. It varies by time of day [24,25] and varies by different levels of congestion [26,27]. Taking dynamic features into account can enhance the accuracy and predictability of accessibility [28,29].

Navigation datasets can be obtained through application programming interface (API) services of online maps based on location-based services (LBS). The datasets contain abundant road information, such as road networks, speed limits, PT stations, traffic status, etc., and can estimate travel times for different transport modes [30]. These datasets are often validated through on-site surveys, making the data more up-to-date and reliable than traditional network analysis [31]. The real-time route planning service API provided by online maps enables estimating various and dynamic travel times by different transport modes under time-variant traffic conditions. Travel data obtained through the online map API is based on the latest road network rather than a self-built road network, which has the advantage of reflecting the real-time traffic status, compared with the traditional network analysis [32]. Recently, numerous studies in terms of traffic accessibility based on online map APIs have emerged. Niu et al. [33] used the Amap API to obtain travel times by walking, driving, and PT to evaluate the accessibility of parks in Wuhan city. Su et al. [34] calculated hospital accessibility at different periods using real-time traffic navigation data obtained by online map API. Chen et al. [35] studied the accessibility of fire stations in Nanjing City using the car travel mode of the Amap API to obtain real-time travel time from fire stations to fire events. Wang et al. [36] analyzed the accessibility of 56 scenic spots in Xi’an City via car and public travel modes using the real-time travel function of the Baidu Map API. These studies have proved that online mapping services can provide objective and accurate travel data. To date, however, current studies are mainly concerned with the accessibility of various public services within the city; the application of online map API is still scarce in the studies on CCA.

In accessibility studies, suitable spatial units are usually selected first for the study area to collect and analyze data. The following two approaches are commonly used to specify spatial units. One approach is to use administrative units such as sub-district [37], census tract [31], community [38], and block [39] as the basic spatial unit, taking their centroids as the origins. The other is to divide the study area into an equal-sized raster, for instance, 1 km × 1 km [40], 500 m × 500 m [33,34,41], 400 m × 400 m [42], with the centroid of each grid as the origin. The latter approach is often used when obtaining travel data using online map APIs. Some scholars [33,37] have highlighted the fact that smaller spatial units can generate better results. When using the online map API to obtain data, higher resolution data can be collected by dividing the study area into grids with smaller units, improving the accuracy of transit accessibility measurements [43]. In this paper, the study area was divided into 100 m × 100 m grids containing 97,605 units in total.

## 3. Data and Methodology

In this section, we introduce the information about the study area relevant to our research and the process of data acquisition. The methods used in the analysis procedure and why we chose GWR are also shown here.

### 3.1. Study Area

**Figure 1:**
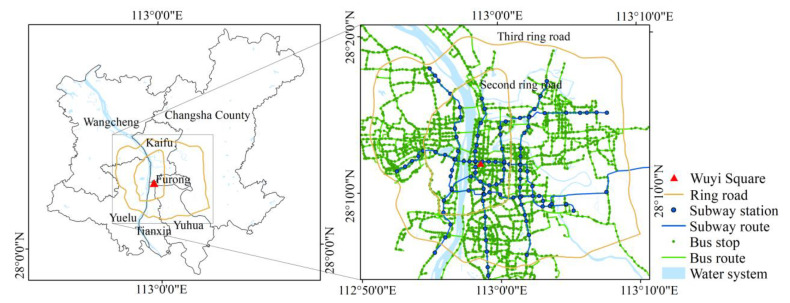
Study area.

Changsha is the capital of Hunan Province, in the south-central part of China, with a total population of up to 10 million in 2020, governing 6 districts and 1 county. In Changsha, there are 6 metro lines, and 102 metro stations, and the average daily metro passenger volume is 1.21 million. There are also 291 bus lines, nearly 5000 bus stops, and the average daily bus passenger volume is 1.3 million. The daytime heat map of Changsha City provided by Baidu Map shows that most population activities are concentrated within the third ring road. Therefore, the area shown in Figure 1 is selected as the study area. Bus lines, metro lines, and ring roads are also plotted in Figure 1 based on data from the Open Street Map. According to the overall plan of Changsha City, Wuyi Square is the center of the city, with a large number of commercial and business lands clustered around it. Meanwhile, Wuyi Square Station is the first transfer station of Changsha Metro, marking the formation of the cross pattern of Changsha Metro.

### 3.2. Travel Data and Population Information

Dividing the study area into grids can be easily combined with grid-based population data to facilitate accessibility modelling, calculation, and evaluation. We divided the study area into 100 m × 100 m grids with 97,605 units (excluding the water system) and obtained the real-time travel data from Baidu Map (https://lbsyun.baidu.com/, accessed on 8 October 2021), one of the biggest online maps in China. The center of each unit is the origin, and the destination is Wuyi square. The route navigation module of Baidu Web Mapping API can provide accurate travel data by PT and automobile from each grid to Wuyi Square based on actual real-time traffic status. A python algorithm program that can access the route navigation module has been used to acquire the necessary data. For an origin-destination trip, the travel data for each mode includes the shortest travel time from the origin to the destination and the corresponding route. The data also contains the actual fare incurred for the PT route and the taxi fare driving along the automobile route. There is no uniform charge for automobiles, and it’s hard to find a proper standard to quantify the cost of using automobiles. Different types (powered by electric, gas, or hybrid) and different driving habits will lead to great variations in driving costs. The cost of purchase, maintenance, insurance, and depreciation for automobiles should also be counted in. Taxi fare is seen as a standardization of the monetary cost of driving to the city center.

Morning and evening peak hours are when traffic congestion on roads and crowding on PT are at their highest. And the frequency of PT service is usually higher during peak hours. To compare the CCA among peak and off-peak hours, we selected three time periods for the study: morning peak (AM), mid-day rest (MD), and evening peak (PM). The data were obtained during the period 11 October 2021–7 November 2021 (four weeks in total) and comprised three hours of a day: AM (8–9 a.m.), MD (1–2 p.m.), PM (6–7 p.m.). Since the travel times obtained are based on real-time road conditions, each origin-destination travel time at different hours is the hour’s average during four weeks to avoid randomness. The demographic data used in this study is the 100 m precision Chinese population data in 2020 provided by WorldPop (https://www.worldpop.org/, accessed on 12 July 2021) and validated with China’s seventh national census data. The population in each spatial unit is obtained by performing a bilinear resampling method in ArcGIS 10.2. All data are carefully checked to avoid abnormal values.

### 3.3. Methods

Our methods are developed as follows. First, utilizing the Baidu online mapping API, we collect the travel time and travel fare from each grid to the city center by automobile and PT. Then we use the travel time to draw the isochrone maps to the city center at AM, MD, and PM for the two transport modes. According to the cumulative opportunity method, we draw the cumulative accessible land and population percentages as travel time increases. Then, we convert the travel time into an equivalent monetary cost based on the average wage in Changsha in 2020 and add it to the actual travel fare to get the travel cost required. Following the method mentioned above, we draw the iso-cost maps and the cumulative curves as travel cost increases. After that, for each unit, the ratio of travel time (time-ratio) and the ratio of travel cost (cost-ratio) for the two transport modes are calculated and visualized. Finally, the variables influencing time-ratio and cost-ratio are explored using the OLS regression and the GWR.

(1) Isochrone map and iso-cost map

Isochrone map provides a visual indication of the area where the city center can be reached within a given travel time. Based on the travel data obtained from the Baidu Map and after the above transformation, each unit in the study area has attributes including travel time and travel cost. And the isochrone map and iso-cost map are generated in ArcGIS 10.2 using the inverse distance weighting (IDW) method with intervals of 10-min and $5. Both isochrone maps and iso-cost maps are drawn for the two travel modes at the three periods (AM, MD, PM), a total of 6 scenarios.

(2) Cumulative opportunities

For the 6 scenarios mentioned above, based on each unit’s travel time or travel cost, we count the land located within a given travel time or cost i, then plot the cumulative growth curves of the land percentage $P(l_{i})$ as the travel time or the cost increases. Combined with each unit’s population information, the cumulative growth curves of the population percentage $P(o_{i})$ are plotted.

(1) $$P(l_{i})=\frac{l_{i}}{L}\times 100\%$$

(2) $$P(o_{i})=\frac{o_{i}}{O}\times 100\%$$

In Equation (1), $l_{i}$ is the land where the city center is accessible within travel time or cost i, L is the total land of the study area, $P(l_{i})$ indicates $l_{i}$ as a percentage of L, while $o_{i}$, O, $P(o_{i})$ in Equation (2) indicate the corresponding population-related indicators.

(3) Regression analysis

For each unit, the ratio of travel time (time-ratio) and travel cost (cost-ratio) to the city center by the two transport modes are calculated and visualized to depict the CCA differences between the two modes. Time-ratio and cost-ratio are used as dependent variables, and possible explanatory variables are analyzed.

(3) $$time-ratio=\frac{travel\;time\;by\;PT}{travel\;time\;by\;automobile}$$

(4) $$cost-ratio=\frac{travel\;cost\;by\;PT}{travel\;cost\;by\;automobile}$$

(5) Y=Xβ+ε

(6) $$Y=X\beta +\varepsilon ,\varepsilon =\lambda W\varepsilon +\mu ,\mu ∼N\left[0,\sigma^{2}I\right]$$

(7) $$Y=\rho WY+X\beta +\varepsilon ,\varepsilon ∼N\left[0,\sigma^{2}I\right]$$

(8) $$y_{i}=\beta_{i0}+\sum_{k=1}^{p}\beta_{ik}x_{ik}+\varepsilon_{i}$$

For an OLS model, the relationship between the dependent variable Y and explanatory variables X can be formulated as Equation (5), where β is the coefficient and ε is the random error. The spatial error model and spatial lag model write as Equations (6) and (7). In Equation (6), Y is an N × 1 vector of dependent observations, X is an N × K matrix of exogenous explanatory variables, β is a vector of coefficients for X, λ is the coefficients of spatial error terms Wε, and μ is the random error. In Equation (7), ρ is the coefficient of lagged dependent observations WY. The regression models that underlie GWR can be written as Equation (8). For the unit i, $\beta_{i0}$ is the constant term of the statistical regression, $\beta_{ik}$ is the regression coefficient of the explanatory variable $x_{ik}$. The GWR model is an extension of the OLS model, and it adds spatial geographic information of the data to the regression parameters.

By using ArcGIS10.2, first, we started fitting our data with OLS (Equation (5)) regression while the error term (ε) is assumed to be at least independent and identically distributed. Cluster and outlier analysis (Anselin Local Moran I) are performed on the residuals of OLS models. Significant positive Moran’s I for the residuals in all models are found, which means the unexplained error term (ε) in OLS models is unlikely independent. The regression analysis of our data should consider the spatial correlation.

Then we fit our models with the spatial error model and spatial lag model using GeoDa. However, the results show that the two models are hard to explain the correlation between variables. When using the spatial error model, the coefficient λ (Equation (6)) of the spatial error term reaches above 0.99; when using the spatial lag model, the coefficient ρ (Equation (7)) of the lagged term also reaches above 0.99, and the residuals of these models are still highly spatially correlated. The spatial error model and spatial lag model are techniques introduced to deal with spatial dependence based on general regression analysis. Although different independent variables have different degrees of influence on the dependent variables, the contribution of individual independent variables is still the same in different areas. Essentially, the two models belong to the global model. In contrast, the GWR can estimate the coefficients for different independent variables at different locations, and the differences in the relationships between variables due to different geographical locations can be detected intuitively. For a spatial phenomenon such as accessibility, it is possible to investigate better the influence of each variable on the spatial variation of accessibility, so we finally choose the GWR.

## 4. Results

In this section, we visualize automobile-based and PT-based CCA by isochrone maps, iso-cost maps, and cumulative curves during the three time periods: morning peak (AM), mid-day rest (MD), and evening peak (PM). Then we present the regression analysis results, cooperating with some reasonable explanation.

### 4.1. Travel Time Measures

**Figure 2:**
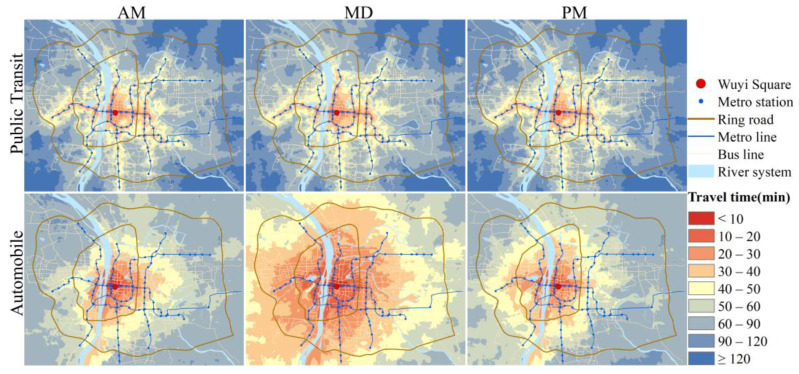
Isochrone maps of automobile and PT for the three time periods.

In terms of spatial distribution (Figure 2), the PT-based isochrone maps show apparent linear expansion along the metro lines, reflecting the shape of the metro network, while the automobile-based maps are more circular, showing a concentric structure that gradually spreads from center to outside. And the “center-edge” gap in PT-based maps is more significant than in automobile-based maps. PT-based isochrone maps waver less with time, while automobile-based maps vary widely between peak and off-peak hours and demonstrate significantly better CCA during MD than the other two periods.

The statistics of travel time by the two modes at the three time periods are shown in Table 1. Both modes exhibit better CCA during MD and show not much difference between morning and evening peak hours; the two modes’ difference is most remarkable during MD. For the entire study area, the mean travel time to access the city center by automobile is 39.5 min, while PT is 80.4 min. The mean time by automobile differs significantly between peak and off-peak hours, with the value being 59.6 min and 57.7 min during peak hours, while 35.3 min during MD, with a decrease of more than 20 min. Travel time by PT varies less among the three time periods. This result is understandable since travel time by PT is less affected by congestion. The bus has scheduled frequency and stops, and its driving speed can not exceed its speed limit even if in good traffic conditions. The bus must stop at each stop, whether there are passengers or not, coupled with the short distance between bus stops (most of the bus stops in the study area are about 500 m apart), making it unlikely to travel at a very high speed. In addition, in the travel routes by PT, part of the journey may require taking the metro, which passes underground and is not affected by traffic congestion.

**Figure 3:**
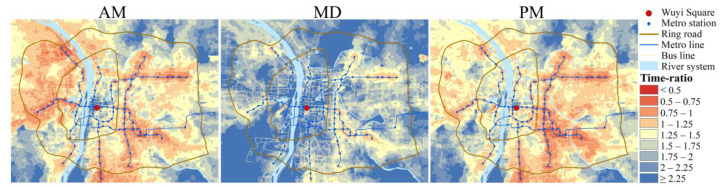
Time-ratio distribution for the three time periods.

Table 1 also shows the mean time-ratio in the study area for the three time periods. The time-ratio equals PT-based travel time divided by automobile-based travel time for each unit (Equation (3)). A unit with time-ratio smaller than one means that it takes less time by PT than by automobile to reach the city center; time-ratio greater than one means the opposite. The time-ratio during MD is higher than during AM and PM. Because the automobile-based travel time decreases significantly at MD, while the PT-based travel time is relatively stable, leading to the time-ratio increases during MD. However, it should be noted that the mean time-ratio during all three periods is greater than one, implying that PT-based CCA is overall inferior to automobile-based CCA from the travel time perspective. The spatial distributions of time-ratio during three time periods are shown in Figure 3. It can be seen that there are fewer areas with time-ratio less than one during MD, and most of them are distributed around metro stations. The more extreme case, where the PT-based travel time is more than double the automobile-based (time-ratio > two), is concentrated in the northeast and southwest of the study area, where bus lines are scarce and there are no metro stations. At peak hours, traffic congestion causes automobile-based travel time to increase in some areas, and more areas appear with time-ratio less than one, but it can still be found that these areas are relevant to metro lines and stations.

**Figure 4:**
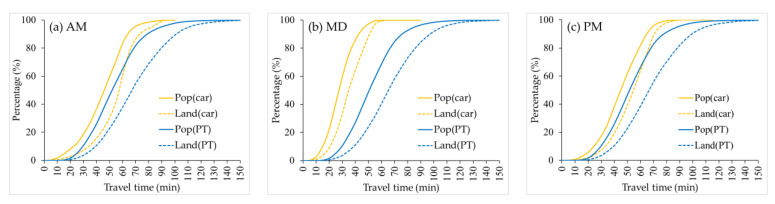
Growth curves of cumulative land and population percentages as travel time increases: (**a**) during AM; (**b**) during MD; (**c**) during PM.

According to the cumulative opportunities method, the number or percentage of a kind of element that can be accessed in a given time can be used to assess accessibility. The cumulative CCA can be indicated by the percentage of land or population which can access the city center. Figure 4 compares PT-based and automobile-based cumulative CCA growth curves for three time periods, showing that all curves exhibit like S-curve. Overall, automobile users have relatively better cumulative CCA than PT users in terms of travel time. In other words, when traveling by automobile, the city center is accessible to a larger proportion of the population and land within a given travel time. The cumulative CCA differences between the two modes are more significant during MD when it seems rather inequitable that the city center is within a 60 min drive by automobile for almost the whole land (99.21%) and (96.12%) population, while just about 30% (28.06%) of the land and 60% (60.83%) of the population within a 60 min drive by PT. However, it should be recognized that even during MD, when there is little congestion, the cumulative CCA is poor for both modes and worse for PT. We can see that the city center is within 15 min drive by PT for just 0.05% land and 0.23% population of the study area, and within 15 min drive by automobile, there are 2.43% land and 8.8% population. And if travel time by PT could cover a similar percentage as the automobile’s 15 min drive, it would take almost 30 min (2.3% land and 8.99% population), double the automobile travel time.

Also, the curves of both modes show that the percentage of population which can access the city center increases faster than that of land when travel time increases. It also refers to that both modes can make the city center accessible for a larger percentage of the population than land within a given travel time. It is a result of the fact that population and land are not uniformly distributed within urban areas. For urban areas, population density tends to decrease gradually from the city center to the suburbs. A certain percentage of land close to the city center carries more than that percentage of the population. Supposing we use the percentage of population served as an indicator to evaluate the cumulative CCA, we will get better results than using the percentage of land, no matter which transport mode is chosen.

### 4.2. Travel Cost Measures

The advantages of PT are also known to be its low fares and relatively stable travel time, and it is biased to use travel time alone to compare the two modes. Therefore, we try to use travel cost to compare the CCA of both modes, considering both travel fare and travel time.

(9) travel cost=(travel time×8.42)+travel fare

**Figure 5:**
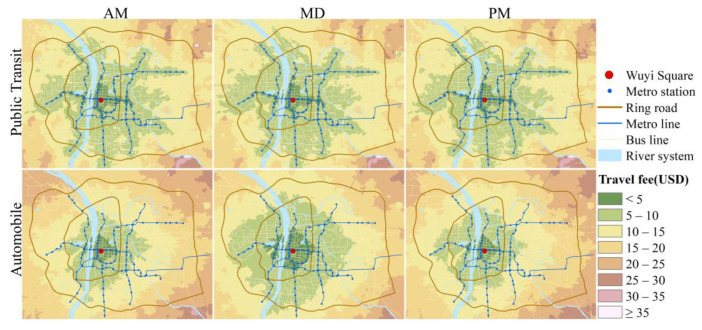
Iso-cost maps of automobile and PT for the three time periods.

In equating travel time to monetary value, some use the minimum wage [44], and some use the average wage [15], the latter is used in this study. The time value of Changsha citizens is about 53.44 yuan ($8.42)/h, calculated based on Changsha’s total employee wages in 2020. The travel time is transferred to monetary value and summed with the actual travel fare as the travel cost required for each unit to reach the city center (Equation (9)). As mentioned in 3.2, the travel fare of PT is the actual fare incurred during the trip, while the travel fare of the automobile is measured using the taxi fare on the same route at the same time. Iso-cost maps are drawn using a $5 interval to observe the spatiotemporal distribution of CCA for the two modes (Figure 5). It can be observed that the spatial distribution of the iso-cost maps is similar to the isochrone maps, i.e., PT-based iso-cost maps expand along the metro lines, and automobile-based show concentric structure.

Table 2 shows the two modes’ mean travel costs to the city center and the cost-ratio (Equation (4)). We can find the travel costs of both modes are lower during MD. And the mean cost by automobile is closer to PT during MD but significantly higher than PT during AM and PM since automobile-based cost increases during peak hours. The mean cost-ratio is higher during MD, but it is less than one for all the three time periods, i.e., the mean travel cost by PT is lower than that by automobile during peak and off-peak hours. Measured from the mean travel cost perspective, traveling by PT has better CCA.

**Figure 6:**
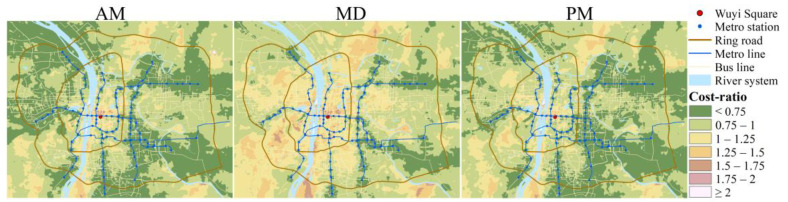
Cost-ratio distribution for the three time periods.

The spatial distribution of cost-ratio (Figure 6) shows that the automobile no longer prevails when travel cost is the CCA measure, especially at peak hours. Cost-ratio in most areas is less than 1, which means at peak hours, there are many areas where the travel cost reaching the city center by PT is lower than that by automobile. Moreover, values of cost-ratio show a trend of gradually decreasing from the center to the periphery, meaning the travel cost advantage of PT is gradually evident from the center to the periphery, and the surroundings of metro stations always have a smaller cost-ratio. During MD, the areas with cost-ratio less than one decrease compared to peak hours, indicating the advantage of PT declines compared with peak hours.

**Figure 7:**
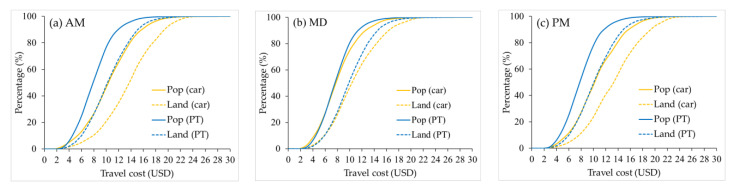
Growth curves of cumulative land and population percentages as travel cost increases: (**a**) during AM; (**b**) during MD; (**c**) during PM.

Similarly, we draw the growth curves of cumulative population and land percentages when travel cost increases (Figure 7). The cumulative curves still show the characteristics of the S-curve. Disparities between population and land still exist in both modes. Unlike the cumulative CCA curves measured by travel time, when using travel cost as the *X*-axis, the PT-based cumulative CCA is mostly better than automobile-based, both in population and land. And differences between the two modes are greater during peak hours and very similar during MD. This finding suggests, to some extent, that there is not a huge accessibility deficit between PT and automobile in Changsha in terms of travel cost when road conditions are good.

### 4.3. Regression Analysis

Clarifying the impact of influencing factors on CCA differences between the two modes can provide suggestions for the construction and layout of PT. Time-ratio and cost-ratio are the ratios of the two modes concerning travel time and travel cost. From the spatial distribution of the two indicators, both in travel time and travel cost, the automobile-based CCA of each unit shows a concentric structure centered on the city center, and the PT-based CCA tends to expand along transport lines and stations. It can be inferred that automobile-based CCA is correlated with the distance from the city center, and PT-based CCA is related to the distance from the transport station, as the transport station is a critical node of accessibility. To make the magnitude of each variable approximate, use “km” as the unit. To minimize the effect of traffic congestion, we choose the time-ratio and cost-ratio during MD as dependent variables. And the distance from the city center (center-dist), bus stops (bus-dist), and metro stations (metro-dist) are used as explanatory variables to investigate the relationship between them.

First, the data are fitted using the OLS regression, and the results shown in Table 3 indicate that the OLS regression can only explain a tiny fraction of the data. Moreover, in an OLS model, the error term is assumed to be at least independent and identically distributed. Moran’s I of the residuals in the two models are 0.86 and 0.84, respectively, so regressions of the data should take spatial correlation into account. As mentioned in Section 3.3, we used the GWR to analyze the relationship between the dependent and explanatory variables. When choosing a travel route, people often walk to it if the metro station is nearby. If the metro station is far, people often walk to a nearby bus stop to take a bus first to reach a nearby metro station. Considering the bus stop spacing is about 500 m, a distance beyond 500 m may cause people to choose different bus stops and thus different travel routes. Two GWR models are constructed with time-ratio and cost-ratio as dependent variables, respectively, with fixed kernel type and fixed bandwidth of 500 m in model construction.

**Figure 8:**
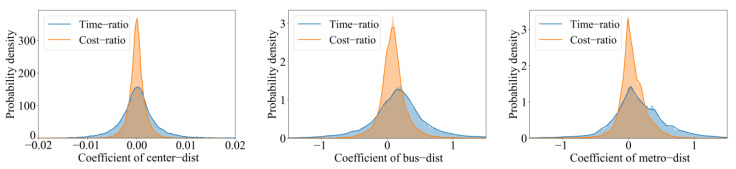
Probability density distribution of the correlation coefficient of each explanatory variable.

The GWR model with time-ratio as the dependent variable has an adjustedR^2^ of 0.82 and an AICc of −10,576.64. In the other model with cost-ratio as the dependent variable, the adjustedR^2^ is 0.81, and the AICc is −90,258.68. It can be seen that the GWR can explain the two models better. The effect of the explanatory variables on the dependent variables in a GWR model is spatially heterogeneous, i.e., the regression coefficient of the explanatory variables varies in different areas. Based on the regression results of the above two GWR models, we draw probability density distributions and positive-negative effects of the regression coefficients for each variable, as shown below (Figure 8 and Table 4). As shown in Figure 8, the coefficient distribution of center-dist shows a relatively standard normal distribution. The bus-dist and metro-dist coefficients show right-skewed patterns, and the right-skewed is more prominent especially when time-ratio is used as the dependent variable. Table 4 shows that center-dist has comparable positive and negative effects on the dependent variables, while bus-dist and metro-dist have mostly positive effects. The farther the unit is from a bus stop or metro station, the larger the time-ratio and cost-ratio may be. Alternatively, PT-based CCA is more likely to be similar or even superior to automobile-based CCA for units near bus stops and subway stations.

**Figure 9:**
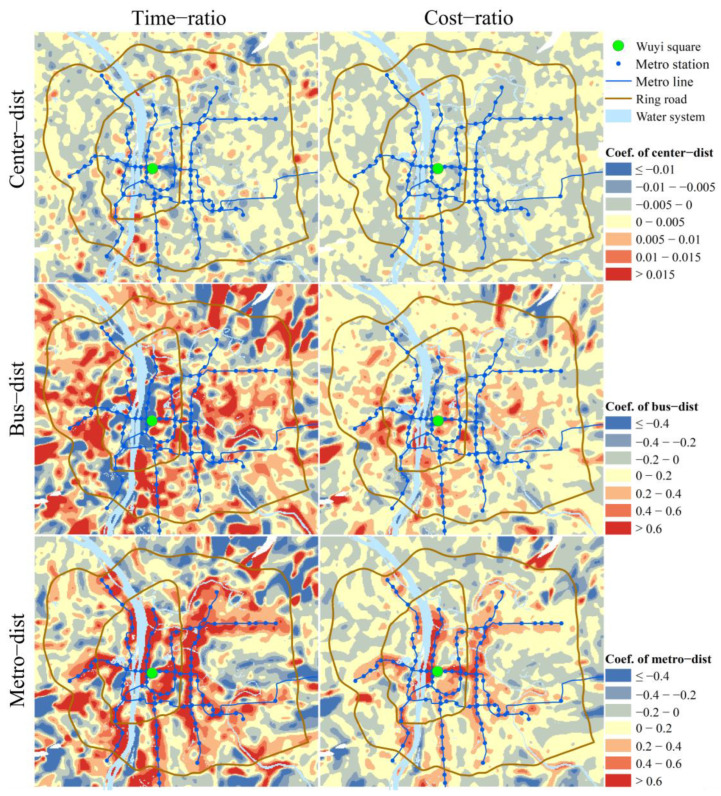
Spatial distribution of the regression coefficients for each explanatory variable.

In the time-ratio and cost-ratio models, each unit has different regression coefficients for the explanatory variables: center-dist, metro-dist, and bus-dist. Figure 9 shows the spatial distribution of the regression coefficients of each variable (according to ArcGIS official documentation, the small blank area is due to the multicollinearity of the explanatory variables in this area, which leads to unreliable results). Compared with the time-ratio model, when cost-ratio is the dependent variable, each explanatory variable’s positive and negative effects become weaker. It reflects the correlation between the explanatory variables and time-ratio is more robust than that with cost-ratio. Table 4 and Figure 9 show that the center-dist’s positive and negative effects are roughly equivalent, and its spatial distribution of positive and negative effects is also random. And the center-dist’s correlation coefficients are small, indicating that the center-dist has weak correlations with time-ratio and cost-ratio. The effect of bus-dist within the second ring road is more complex, with both positive and negative effects distributed and to a high degree, while between the second ring and the third ring road is dominated by positive effects with a moderated degree. Outside the third ring road, there are highly positive and high negative correlation areas. When referring to the metro-dist, the positive effect is concentrated around metro stations. Both time-ratio and cost-ratio models show this trend, and the difference is that the degree and scope of the positive effect around metro stations are smaller in the cost-ratio model.

**Figure 10:**
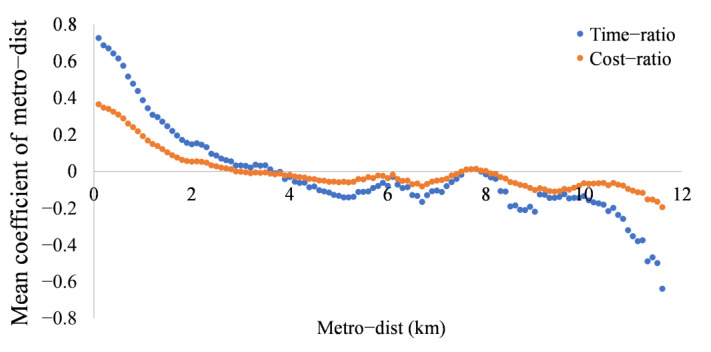
Mean coefficient of metro-dist for units within the same range from the metro stations.

Concerning the scope of effects, we counted the correlation coefficients of the units within the same distance range from metro stations. For units with the same range of metro-dist (100 m interval), their mean correlation coefficients of metro-dist are calculated, as shown in Figure 10. We can find that when time-ratio and cost-ratio are used as the dependent variables, the correlation coefficient decreases with increasing metro-dist, and the closer the distance to metro stations, the stronger the positive correlation. In which, when time-ratio is the dependent variable, the mean value of the correlation coefficient drops below zero when metro-dist exceeds 3.6 km. In other words, the positive effect of metro-dist on time-ratio is mainly concentrated in the area within 3.6 km of metro stations. Although some areas have negative effects within this scope, the positive effect is dominant overall. The corresponding distance is 2.8 km when cost-ratio is the dependent variable, i.e., the mean value of the correlation coefficient drops below zero when metro-dist exceeds 2.8 km.

## 5. Discussion and Conclusions

This paper uses the real-time travel data captured via the Baidu Map API to visualize the automobile-based and PT-based spatiotemporal CCA at a 100 m × 100 m spatial resolution and compare them from travel time and cost perspectives. We focused on the CCA differences between the two modes, quantified the differences, and explored the possible influencing factors. Findings from our analysis are outlined and discussed as follows.

Firstly, we obtain the travel time and the actual travel fare of each unit in the study area to reach the city center and calculate the equivalent travel cost of each unit by converting the travel time into its monetary value using the average time value. Based on this, isochrone maps and iso-cost maps are made for the two modes at different hours. The results show that automobile-based CCA has significant temporal characteristics, with it being better during MD than peak hours, while PT-based CCA differences between peak and off-peak hours are pretty slight. Automobile-based CCA shows a concentric structure centered on Wuyi Square, and PT-based CCA has noticeable expansion along the metro lines, and these spatial characteristics exist at peak and off-peak hours. This indicates that automobile-based CCA is greatly influenced by road conditions. Due to bus and metro operating rules, PT-based CCA is little affected by traffic congestion. According to the metro scheduling rules in the study area, the metro departure interval is even shortened during the morning and evening peak hours to reduce the waiting time of passengers and transport more users. All these together lead to a slight difference between peak and off-peak hours in the PT-based CCA and a greater spatial dependence on the distribution of transport stations. And this is in line with what many researchers have pointed out: transport stations are virtual nodes in the study of PT accessibility.

Secondly, to consider the fare advantage of PT and to avoid the one-sidedness of using only travel time to measure accessibility, this study uses both travel time and travel cost to measure CCA. In summary, the CCA gaps between the two modes will be lessened, and the advantage of the automobile will no longer be evident if using the travel cost instead of travel time as the measurement. When measured by travel time, automobile-based CCA exhibits better, and the CCA difference between the two modes is more significant at MD hour. When measured by travel cost, PT shows better CCA, and the differences differ greater at peak hours when the travel cost of PT is lower than that of the automobile in most areas. The cumulative CCA of the two modes is measured by the cumulative accessible land and population and exhibits similar results to CCA. The automobile always makes the city center accessible for more population and greater land for a given travel time. In comparison, PT is the one that brings more population and land for a given travel cost. An attractive observation is that the two modes’ cumulative CCA at MD hour are very similar when measured by travel cost, indicating that PT is relatively well developed from the travel cost perspective. And there is no huge travel cost inequality between it and the automobile during off-peak hours.

Finally, we quantified the CCA differences between the two modes, and considering accessibility is a spatial phenomenon, we chose the GWR to analyze the factors influencing the CCA differences. According to the spatial distribution characteristics of automobile-based CCA and PT-based CCA, the relevant factors affecting the CCA differences are summarized: center-dist, bus-dist, and metro-dist. The time-ratio and cost-ratio at MD hour (reducing the interference of traffic congestion) are used as dependent variables. Each unit’s center-dist, bus-dist, and metro-dist are explanatory variables. We find that the center-dist of each unit has a negligible effect on the CCA differences between the two modes. The positive effects of bus-dist and metro-dist on CCA differences are mainly observed, and the distribution of bus-dist’s positive effects does not show prominent spatial characteristics. The positive effects of metro-dist are mainly distributed around the metro stations, concentrated within a 3.6 km scope in the time-ratio model and a 2.8 km scope in the cost-ratio model. And in these scopes, the average correlation coefficient decreases with the increase of metro-dist. It suggests that the closer the unit to the metro station, the more likely it has a better PT-based CCA. When developing strategies to improve PT, it may be more efficient to appropriately increase construction density and population or job density around metro stations than to build new transportation infrastructures, considering that the building process is costly and time-consuming.

Measuring spatiotemporal accessibility via an online map API is rapidly evolving and can be expected to be more widely used in the future. The detailed information contained in the real-time travel data also provides the basis for a multi-dimensional assessment of accessibility. This paper is a meaningful attempt to use dynamic data for urban analysis. Compared to using road network data and road average speed for accessibility evaluation, real-time data has a natural advantage in analyzing temporal characteristics. This paper focuses on the comparison of CCA of PT and automobile. At the same time, the online map API also provides route planning for other modes of travel, such as cycling and walking, which can also provide support for the comparison of more travel modes. The approach adopted in this paper can quantify the accessibility of any other valued destinations using other transport modes in the urban area, depending on the research purpose.

Moreover, some studies [45,46] underline that geospatially objective accessibility and perceived subjective accessibility do not exactly match, and individuals’ perspectives should be considered in further research. This study does not capture the traveler preferences, nor does it consider the proportion of users who choose driving or using PT. Also, in general, the time value of the PT user should be inconsistent with the automobile user, which may affect the results of accessibility assessed by travel cost. These questions need to be further explored in future studies.

## Figures and Tables

**Table 1:** Travel time by two modes and time-ratio for the three time periods.

	Time by Automobile (Min)			Time by PT (Min)			Time-Ratio		
	AM	MD	PM	AM	MD	PM	AM	MD	PM
mean	59.6	39.5	57.7	83.1	80.4	82.5	1.41	2.07	1.44
std	15.4	12.4	15.1	33.9	32.3	33.5	0.61	0.88	0.78

**Table 2:** Travel cost by two modes and cost-ratio for the three time periods.

	Cost by Automobile (USD)			Cost by PT (USD)			Cost-Ratio		
	AM	MD	PM	AM	MD	PM	AM	MD	PM
mean	15.6	12.8	15.4	12.5	12.1	12.3	0.80	0.97	0.81
std	5.0	4.9	5.1	4.9	4.7	4.9	0.20	0.22	0.19

**Table 3:** Results of the OLS regression.

DV	Time-Ratio			Cost-Ratio		
	Coef	StdError	Prob	Coef	StdError	Prob
(Intercept)	1.819	0.0046	****	0.995	0.002	****
Bus-dist	0.055	0.0013	****	0.005	0.0006	****
Metro-dist	0.056	0.0009	****	0.002	0.0004	****
Center-dist	−0.001	0.0004	****	−0.003	0.0001	****
AdjR^2^	0.134			0.005		
AICc	145,012.807			−25,578.091		

Significance: **** < 0.001. Corrected Akaike information criterion (AICc) is a way of selecting a model from a set of models, and a lower AICc means a better model.

**Table 4:** The percentage of positive and negative effects of each explanatory variable.

	Time-Ratio			Cost-Ratio		
	Center-Dist	Bus-Dist	Metro-Dist	Center-Dist	Bus-Dist	Metro-Dist
Positive effect (%)	51.11	69.84	65.10	49.62	71.27	61.81
Negative effect (%)	48.89	30.16	34.90	50.38	28.73	38.19

## Data Availability

Not applicable.
